# BUB1B and circBUB1B_544aa aggravate multiple myeloma malignancy through evoking chromosomal instability

**DOI:** 10.1038/s41392-021-00746-6

**Published:** 2021-10-07

**Authors:** Xiaozhu Tang, Mengjie guo, Pinggang Ding, Zhendong Deng, Mengying Ke, Yuxia Yuan, Yanyan Zhou, Zigen Lin, Muxi Li, Chunyan Gu, Xiaosong Gu, Ye Yang

**Affiliations:** 1 Nanjing Hospital of Chinese Medicine affiliated to Nanjing University of Chinese Medicine, Nanjing, China; 2 School of Medicine & Holistic Integrative Medicine, Nanjing University of Chinese Medicine, Nanjing, China; 3 School of Pharmacy, Nanjing University of Chinese Medicine, Nanjing, China; 4 Key Laboratory of Neuroregeneration of Jiangsu and Ministry of Education, Nantong University, Nantong, China

**Keywords:** Haematological cancer, Non-coding RNAs

## Abstract

Multiple myeloma (MM) is an incurable plasma cell malignancy in the bone marrow characterized by chromosome instability (CIN), which contributes to the acquisition of heterogeneity, along with MM progression, drug resistance, and relapse. In this study, we elucidated that the expression of BUB1B increased strikingly in MM patients and was closely correlated with poor outcomes. Overexpression of BUB1B facilitated cellular proliferation and induced drug resistance in vitro and in vivo, while genetic targeting BUB1B abrogated this effect. Mechanistic studies unveiled that enforced expression of BUB1B evoked CIN resulting in MM poor outcomes mainly through phosphorylating CEP170. Interestingly, we discovered the existence of circBUB1B_544aa containing the kinase catalytic center of BUB1B, which was translated by a circular RNA of BUB1B. The circBUB1B_544aa elevated in MM peripheral blood samples was closely associated with MM poor outcomes and played a synergistic effect with BUB1B on evoking CIN. In addition, MM cells could secrete circBUB1B_544aa and interfere the MM microenvironmental cells in the same manner as BUB1B full-length protein. Intriguingly, BUB1B siRNA, targeting the kinase catalytic center of both BUB1B and circBUB1B_544aa, significantly inhibited MM malignancy in vitro and in vivo. Collectively, BUB1B and circBUB1B_544aa are promising prognostic and therapeutic targets of MM.

## Introduction

Multiple myeloma (MM) is a molecularly and cytogenetically heterogeneous hematological malignancy that originates in the bone marrow (BM). Despite the targeted drugs, such as immune modulators and proteasome inhibitors, have greatly improved the outcome of MM patients over the decades, MM remains life-threatening and incurable.^1,2^ It is well known that genetic and epigenetic aberrations, clonal heterogeneity, and clonal evolution play indispensable roles in MM progression, drug resistance and relapse. However, the molecular basis of MM pathogenesis still has not been fully understood. Our studies pointed out that chromosomal instability (CIN) accelerated the development of MM malignancy and drug resistance, leading to the treatment failure and relapse, which limited the effectiveness of most current therapies.^3,4^ Hence, identification of novel molecules and signaling pathways involved in the relationship between CIN and MM is fundamentally essential.

BUB1 mitotic checkpoint serine/threonine kinase B (BUB1B) is a conserved multifunctional protein that is vital for the function of mitotic spindle checkpoint and correcting kinetochore-microtubule attachments.^5^ Inactivation of BUB1B has been confirmed to result in both loss of the spindle checkpoint and severe chromosome segregation defects.^6^ It has been reported that broad elevation of BUB1B is closely associated with high cell proliferation and poor clinical outcome among various kinds of cancers including MM.^7–9^ By employing sequential gene expression profiling (GEP) in MM patient samples, our group firstly pointed out that BUB1B induced CIN in MM.^10^ However, the precise mechanism of BUB1B-mediated promotion of MM remains dimness.^10^

MM is highly dependent on the BM microenvironment, and the interaction of MM counterpart cells and other cells in the BM is of great importance to the development of MM therapeutics. The advanced achievement on the research of circular RNAs (circRNAs) inspires us to explore if the across talk of drug resistance and malignance within the MM counterpart cells is partly by the circRNAs shuttle. Circular RNAs are a fascinating class of single-stranded closed RNA molecules that are derived from precursor mRNA back-splicing or skipping events throughout the eukaryotic genome, forming covalently closed continuous loops. Particularly, circRNAs have been elucidated to play crucial roles in cancer initiation, development, invasion, and drug resistance.^11^ Importantly, circRNAs exert an essential impact on the tumor microenvironment through intercellular communication ascribed to their abundance in exosomes and human fluids.^12^ Therefore, circRNAs are now being visualized as promising biomarkers for cancers.^13^

In this study, we not only characterized the contributing role of BUB1B on MM cell proliferation and drug resistance, but also identified that the circular form of BUB1B gene encodes a novel 544-amino acid protein in MM cells, termed as circBUB1B_544aa. Interestingly, circBUB1B_544aa contains BUB1B kinase catalytic center and could be potentially secreted into the BM microenvironment, the potential role of which in MM was investigated in vitro and in vivo. Clinical samples and patient data were also adopted to evaluate the relationship between BUB1B or circBUB1B_544aa expression and patient outcomes. Finally, we demonstrated a novel downstream target of BUB1B and circBUB1B_544aa. These findings provide significant insights into the functional importance of BUB1B and circBUB1B_544aa as promising prognostic and therapeutic targets of MM.

## Results

### Heightened expression of BUB1B is correlated with poor survival in MM

**Fig. 1:**
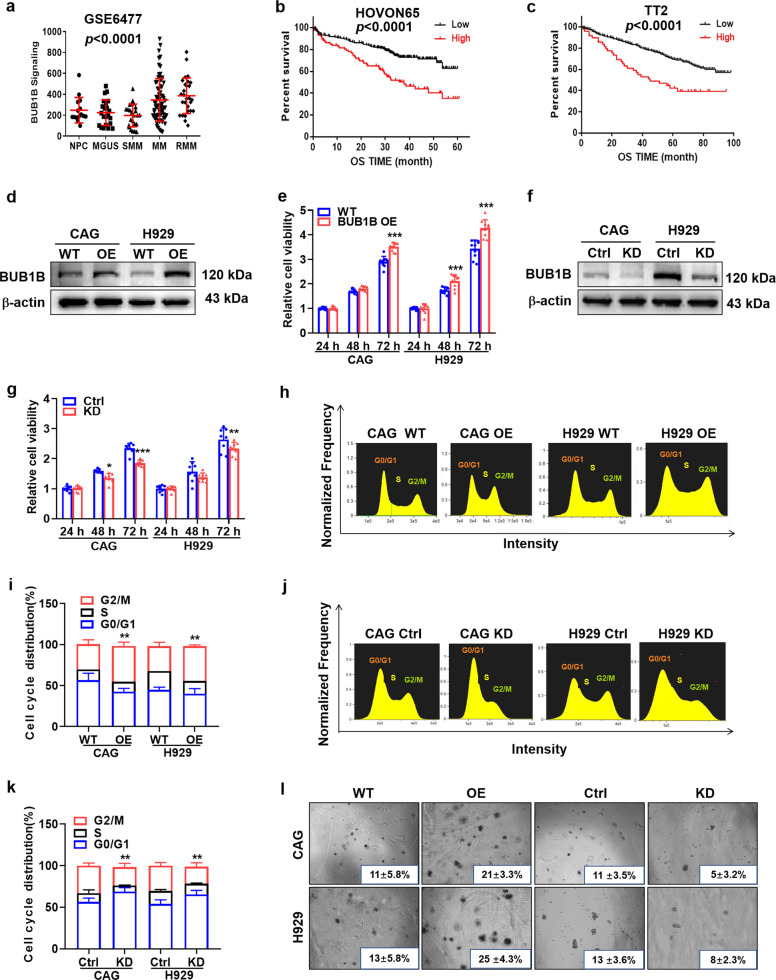
Elevated BUB1B confers poor survival of MM patients and promotes MM cell proliferation. **a** BUB1B mRNA levels were significantly elevated in MM patients. The signal level of BUB1B was shown on the *y*-axis. Patients were designated as healthy donors with normal bone marrow plasma cells (NP, *n* = 15), monoclonal gammopathy of undetermined significance (MGUS, *n* = 22), smoldering multiple myeloma (SMM, *n* = 24), multiple myeloma (MM, *n* = 69), or relapse multiple myeloma (RMM, *n* = 28), are sorted on the *x*-axis. **b**, **c** Increased BUB1B mRNA expression was positively associated with poor overall survival (OS) in MM patients from HOVON65 (**b**) and TT2 (**c**) patient cohorts. **d** Enforced BUB1B expression was detected by WB in CAG and H929 BUB1B-OE cells relative to WT cells. **e** High BUB1B expression facilitated cell proliferation in CAG and H929 cells. **f** Confirmation of BUB1B protein knockdown (KD) in CAG and H929 cells upon transfection with BUB1B-targeting shRNAs. **g** Depletion of BUB1B in MM cells blunted cell proliferation in CAG and H929 cells. **h**–**k** The distribution of different cell cycle phases in WT, BUB1B-OE and BUB1B-KD cells was determined by flow cytometry. Cell cycle analysis revealed that the proportion of G2/M phase significantly increased in BUB1B-OE cells (**h, i**) and decreased in BUB1B-KD cells (**j**, **k**) relative to control cells. **l** Images of representative soft agar plates, revealing accelerated clonogenic growth of BUB1B-OE cells and suppressed clonogenic growth of BUB1B-KD cells compared to WT cells. The data are expressed as mean ± SD. *p < 0.05, **p < 0.01, ***p < 0.001

To explore the role of BUB1B in MM, the GEP dataset of normal plasma (NP), monoclonal gammopathy of undetermined significance (MGUS), smoldering multiple myeloma (SMM), multiple myeloma (MM), and relapse multiple myeloma (RMM) were analyzed initially. BUB1B expression was significantly elevated in plasma cells from MM (*n* = 69) and RMM (*n* = 28) patients, as compared to NP (*n* = 15), MGUS (*n* = 22) and SMM (*n* = 24) (*p* < 0.0001) (Fig. 1a). Notably, the expression of BUB1B was evidently associated with inferior outcome in HOVON65 (*p* < 0.0001) (Fig. 1b) and TT2 (*p* < 0.0001) (Fig. 1c) patient cohorts, suggesting that BUB1B is a potential biomarker of MM poor prognosis.

### BUB1B promotes MM cell proliferation and clonal expansion

To determine whether BUB1B functions as a real driver gene for MM cellular proliferation, BUB1B was overexpressed (OE) in MM cells through lentiviral transfection, as validated by WB (Fig. 1d). The MTT assay showed that the proliferation rate of CAG and H929 cells was significantly increased in BUB1B-OE cells in a time-dependent manner (*p* < 0.001) (Fig. 1e). Inversely, BUB1B was knocked down (KD) by BUB1B-targeting shRNAs, and WB was performed to verify the KD efficiency in both CAG and H929 cells (Fig. 1f). BUB1B-KD MM cells displayed significantly lower cell growth rate than the control (Ctrl) cells (*p* < 0.01) (Fig. 1g). These results showed that BUB1B facilitated MM cellular proliferation. Besides, cell cycle analysis demonstrated that enforced expression of BUB1B elicited prominent increment of G2/M phase proportion (*p* < 0.01) (Fig. 1h, i), while attenuated expression of BUB1B resulted in the remarkable decreased proportion of G2/M phase in MM cells (*p* < 0.01) (Fig.1j, k). Consistently, a clonogenic soft agar assay demonstrated that OE or KD BUB1B significantly switched the long term self-renewal of MM cells, which were evidenced by the apparent increased or diminished clonal formation in both CAG and H929 cells, respectively (Fig. 1l). These findings further support the contention that BUB1B stimulates MM cellular growth.

### Upregulation of BUB1B induces drug resistance in MM

**Fig. 2:**
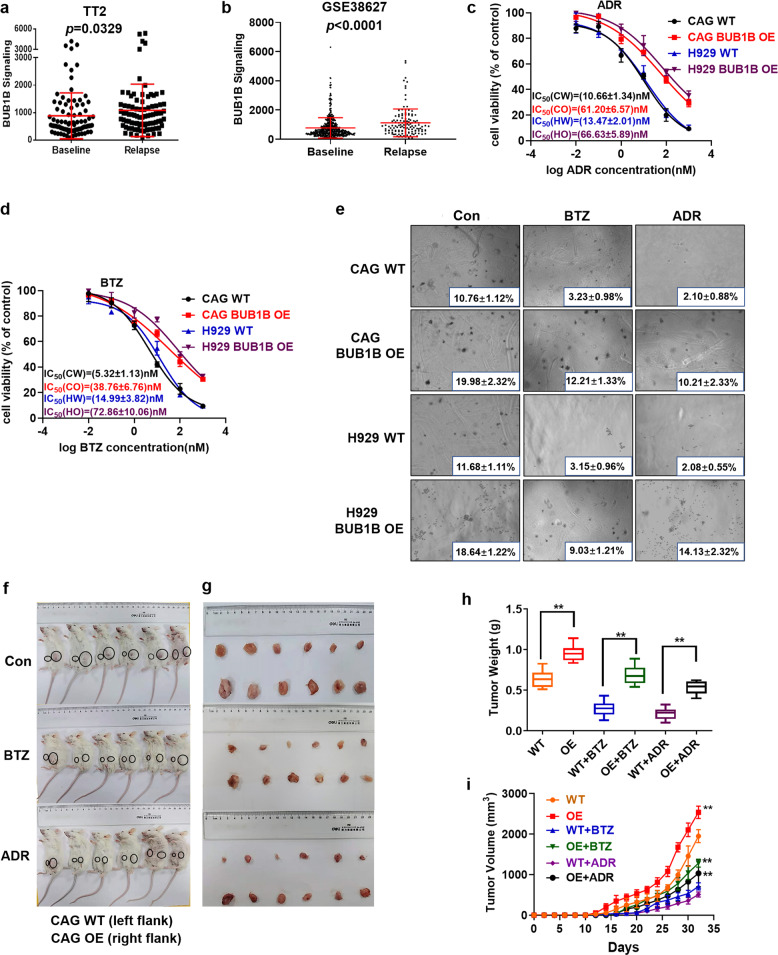
BUB1B induces MM drug resistance. **a**, **b** Box plot represented that elevated BUB1B was observed in the relapsed MM patients compared to that at baseline from TT2 (**a**) and GSE38627 (**b**) patient cohorts. **c**, **d** Effects of BTZ and ADR on the cell viability of CAG and H929 cells. WT cells exhibited more sensitive to BTZ and ADR treatment than BUB1B-OE cells. **e** Images of representative soft agar plates. Colony efficiency values decreased significantly in the control group, but decreased much less in BUB1B-OE group upon drug treatment. **f** Photographic images of xenograft-bearing mice from each group were captured. **g** Photographic images of xenografts were taken from NOD-SCID mice of each specified group. **h** Mean tumor weights in the six experimental groups at day 32 after implantation of the specified MM cells. **i** Time course of tumor growth in NOD-SCID mice treated with vehicle, BTZ, or ADR. The data are the mean ± SD. *p < 0.05, **p < 0.01, ***p < 0.001

Since our previous study have demonstrated BUB1B was enriched in the high-risk MM subgroups, which was closely associated with relapse and drug resistance, we further measured BUB1B expression in relapse MM samples. The analysis showed that elevated BUB1B expression was observed in relapse MM samples from TT2 and GSE38627 cohorts compared to the newly diagnostic counterparts (*p* *<* 0.05) (Fig. 2a, b). To further explore the correlation between BUB1B and drug resistance, we adopted MTT assay to evaluate the IC_50_ of BTZ and ADR on BUB1B WT and OE cells, respectively. The IC_50_ values of both BTZ and ADR significantly increased in BUB1B-OE cells compared to WT cells (*p* < 0.001) (Fig. 2c, d). Moreover, a clonogenic soft agar assay was utilized by using BUB1B OE and WT cells (CAG & H929) treated with ADR and BTZ. Compared to the non-treated controls, BUB1B-OE cells showed obviously more clonal formation than WT cells upon ADR and BTZ treatment at the same concentrations (Fig. 2e). To extend these observations in vivo, CAG BUB1B WT or OE cells were injected subcutaneously into the right or left flanks of NOD-SCID mice treated with ADR or BTZ respectively. After 32 days, we observed that tumors derived from BUB1B*-*OE cells visibly grew faster than tumors derived from WT cells (Fig. 2f, g), meanwhile the mean weight and volume were also significantly higher than their counterparts (Fig. 2h, i). Additionally, WT tumors treated with ADR or BTZ lagged behind their corresponding non-treated controls. However, there was no significant difference in the tumor growth rate after treating with the above drugs in the BUB1B-OE group (Fig. 2h, i). Hence, it was indicated that BUB1B-OE cells exhibited potential resistance to both ADR and BTZ treatment in vivo, while WT cells were sensitive to the treatment of ADR or BTZ. The data strongly support that BUB1B is a powerful factor in promoting drug resistance in MM.

### BUB1B evokes chromosomal instability (CIN) through activating CEP170 in MM

**Fig. 3:**
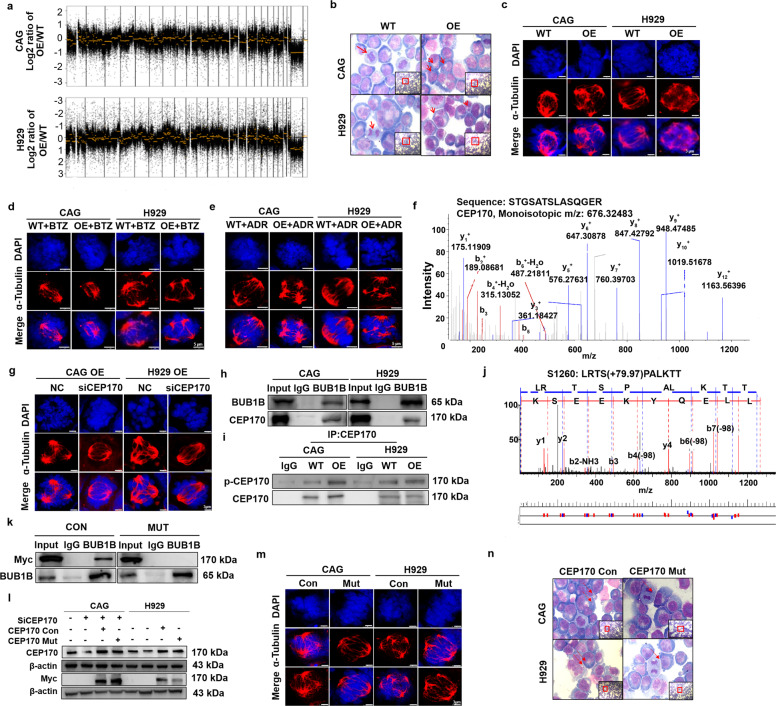
BUB1B evokes MM chromosomal instability (CIN) through activating CEP170. **a** A comparative genomic hybridization (CGH) array revealed significant gains and losses of multiple chromosomal segments in CAG and H929 BUB1B-OE cells relative to WT cells. **b** Giemsa staining showed that BUB1B OE remarkably increased the separation error rate and number of multi-nuclear cells in CAG and H929 cells. **c** Apparent increased chromosomal plate width and decreased mitotic bipolar spindle length were observed in CAG and H929 BUB1B-OE cells relative to WT cells, as demonstrated by immunofluorescent (IF) staining for α-tubulin and DAPI. The scale bar is 3 μm. **d**, **e** In WT and BUB1B-OE cells treated with vehicle, BTZ or ADR, chromosomal plate width was higher and mitotic spindle length was lower in the BTZ and ADR treated BUB1B-OE group compared to WT group. The scale bar is 5 μm. **f** CEP170 was selected among candidate genes of the CIN-related gene list and proteins interacted with BUB1B through mass spectrometry (MS) analysis, which was associated with poor outcome in TT2 patient cohort. **g** Targeting CEP170 by siRNA in CAG and H929 BUB1B-OE cells significantly decreased chromosomal plate width and increased mitotic bipolar spindle length. The scale bar is 3 μm. **h** Physical interaction between BUB1B and CEP170 was confirmed by using Co-IP assay in CAG and H929 BUB1B-OE cells. **i** A Co-IP assay confirmed that BUB1B physically interacted with and phosphorylated CEP170 in BUB1B-OE cells compared to WT cells. **j** MS was employed to determine the BUB1B phosphorylation site of CEP170, Ser1260. **k** A Myc-tagged CEP170 Ser1260Ala mutant exhibited dramatically decreased interaction with BUB1B, as demonstrated by Co-IP followed by WB. **l** Overexpression of native and mutated CEP170 upon endogenous CEP170 was knocked down in CAG and H929 cells. **m** Overexpression of mutated CEP170 Ser1260Ala decreased chromosomal plate width and increased mitotic bipolar spindle length in CAG and H929 cells, as examined by IF staining for α-tubulin and DAPI. The scale bar is 3 μm. **n** Giemsa staining showed that overexpression of mutated CEP170 Ser1260Ala reduced the numbers of multiple nuclear cells. The data are the mean ± SD. *p < 0.05, **p < 0.01, ***p < 0.001

For BUB1B participates in the regulation of the mitotic spindle checkpoint (SAC) to ensure the correct separation of chromosomes during the cleavage process to maintain genome stability,^14,15^ we aim to establish whether BUB1B promotes MM CIN to further elucidate the role of BUB1B in MM biology. We first conducted a comparative genomic hybridization (CGH) array to evaluate the effect of BUB1B on MM chromosomal composition at genomic level. The data identified that significant gains and losses of multiple chromosomal segments in BUB1B-OE CAG and H929 cells relative to the corresponding WT cells (Fig. 3a). Next, pathological Giemsa staining demonstrated that overexpression of BUB1B profoundly promoted two indicators of CIN,^16,17^ numbers of multiple nuclear cells (*p* *<* 0.05) as well as the separation error rate in MM cells (Fig. 3b, Supplementary Fig. 1a). Thirdly, chromosomal plate width and mitotic bipolar spindle length, another two key features of CIN^18,19^ were examined by immunofluorescent (IF) staining for α-Tubulin and DAPI, and it was shown that upregulation of BUB1B remarkably increased chromosomal plate width (*p* *<* 0.05) and decreased mitotic bipolar spindle length (*p* *<* 0.001) in MM cells (Fig. 3c, Supplementary Fig. 1b), revealing that amplification of BUB1B promoted CIN. As CIN is tightly correlated with drug resistance in cancer, we evaluated whether BUB1B OE could overcome sensitivity to BTZ or ADR in MM cells. IF staining assay demonstrated that upon BTZ or ADR treatment, BUB1B OE caused apparent augments in chromosomal plate width and reductions in mitotic spindle length relative to WT cells (*p* *<* 0.05) (Fig. 3d, e, Supplementary Fig. 1c, d). The above data provide the evidence that BUB1B-induced CIN contributes greatly to drug resistance in MM.

To elucidate the mechanism by which BUB1B affected CIN, Co-IP analysis followed by mass spectrometry (MS) was conducted to detect the BUB1B downstream targets, and Centrosomal Protein 170 (CEP170) was identified among the top of the detected proteins interacting with BUB1B (Fig. 3f). CEP170 is a centrosomal component responsible for centrosome microtubule anchoring.^20,21^ Physical interaction between BUB1B and CEP170 was confirmed by using a Co-IP assay in BUB1B-OE CAG and H929 cells (Fig. 3h). In addition, targeting CEP170 by siRNA in BUB1B-OE CAG and H929 cells decreased the CIN features caused by BUB1B, indicating that BUB1B induced MM CIN by directly interacting with CEP170 (Fig. 3g).

As BUB1B functions as kinase, we assumed that BUB1B could phosphorylate CEP170. Co-IP analysis confirmed our assumption and showed that the phosphorylated form of CEP170, detected by anti-phospho-serine antibody, was increased in BUB1B-OE CAG and H929 cells compared to WT cells (Fig. 3i). What’s more, MS analysis identified that CEP170 Ser1260 was the phosphorylation site of BUB1B (Fig. 3j). To further prove that CEP170 Ser1260 was the BUB1B phosphorylation site, we mutated Ser1260 to Ala. The interaction between mutant Ser1260Ala CEP170 and BUB1B was significantly repressed compared to the controls (Fig. 3k). We further transfected WT and mutated CEP170 plasmids into MM cells in which endogenous CEP170 was knocked down, as detected by WB (Fig. 3l). Intriguingly, we found that mutated Ser1260Ala CEP170 could affect CIN, as indicated by decreased chromosomal plate width and increased mitotic spindle length compared to WT cells (Fig. 3m, Supplementary Fig. 1e). In addition, Giemsa staining assay indicated that the numbers of multiple nuclear cells in Ser1260Ala mutant CEP170 OE cells were decreased compared to WT cells (Fig. 3n, Supplementary Fig. 1f, g). Taken together, these findings unveil that BUB1B induces MM CIN by phosphorylating CEP170 at the Ser1260 site.

### CircBUB1B_544aa is identified as a circular RNA with its protein-coding ability

**Fig. 4:**
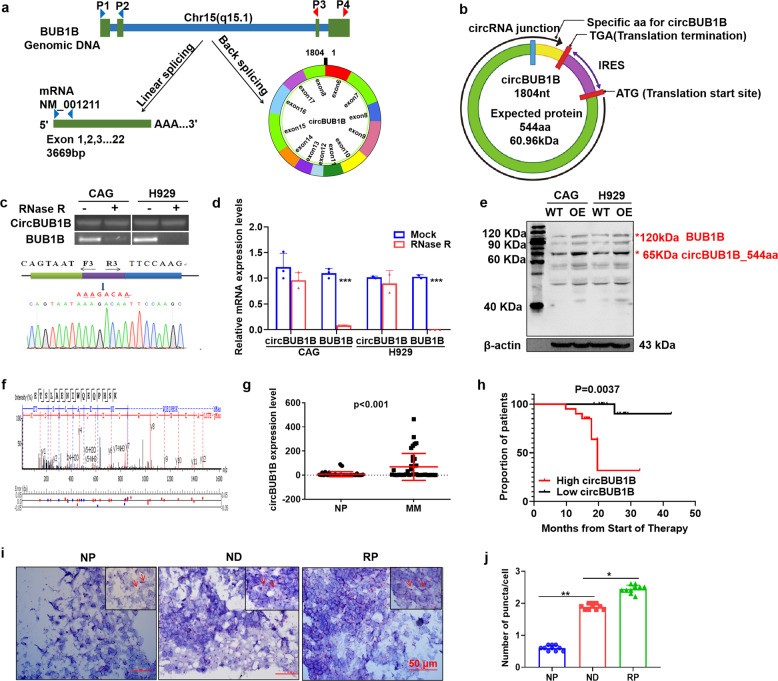
MM cells secrete circBUB1B_544aa to induce CIN and influence cells in the bone marrow microenvironment. **a** Illustration of the annotated genomic region of BUB1B, the putative different RNA splicing forms, and the validation strategy for circular exon 6–18 (circBUB1B). **b** CircBUB1B contained the putative open reading frame (ORF). The sequences of the putative ORF were shown in green, internal ribosomal entrance site (IRES) sequences were shown in purple, and specific amino acid sequences of circBUB1B_544aa were shown in yellow. **c**, **d** The mRNA levels of circBUB1B and linear BUB1B ± RNase R were determined by RT-PCR and qRT-PCR. The number of exons and exact circBUB1B sequences produced from BUB1B were validated by Sanger sequencing. The blue arrow represented the “head-to-tail” splicing sites of circBUB1B. **e** WB analysis of circBUB1B_544aa expression in CAG and H929 BUB1B-OE cells. **f** The protein samples from CAG and H929 cells were subject to mass spectrometry analysis. The specific peptides from circBUB1B_544aa were identified. **g** circBUB1B levels were significantly elevated in MM patients. **h** Elevation of circBUB1B expression was associated with inferior EFS survival. **i**, **j** The levels of circBUB1B in relapse patients (RP) patients were superior than newly diagnosed (ND) patients and normal control (NP) evaluated by RNA scope analysis, and the representative staining images were shown while positive reactions were indicated by arrows. The scale bar is 50 μm. The data are the mean ± SD. *p < 0.05, **p < 0.01, ***p < 0.001

The bone marrow microenvironment is vitally important to the oncogenic growth of MM cells; further studies have been performed to explore the impact of circular RNAs on the MM microenvironment.^12^ We first predicted the presence of a secreted circBUB1B circular RNA fragment (1804 bp) containing 13 exons using circbase database (Fig. 4a), which contained a putative internal ribosome entry site (IRES) sequence and might encode a novel BUB1B isoform with 544 amino acids (Fig. 4b), termed as “circBUB1B_544aa” in the present study. To confirm that exons 6 and 18 of the BUB1B gene formed an endogenous circRNA, convergent and divergent primer were designed to detect linear form of BUB1B mRNA and the circular form, respectively. Upon RNase R digestion, linear form was significantly degraded (*p* *<* 0.001), while circBUB1B exhibited resistance, approving the existence of circBUB1B (Fig. 4c, d). Next, Sanger sequencing recognized the circBUB1B junction site (Fig. 4c), which confirmed the presence of circBUB1B additionally. Then, we exploited a BUB1B antibody to successfully identify that circBUB1B_544aa was elevated in CAG and H929 BUB1B-OE cells as expected, which specifically recognized the N-terminus of BUB1B (Fig. 4e). MS analysis further validated the specific peptide fragments from circBUB1B_544aa (Fig. 4f). Collectively, our data demonstrate the presence of a secreted circBUB1B RNA fragment and confirm the protein-coding ability of circBUB1B, termed as circBUB1B_544aa.

To strengthen our findings in MM primary samples, we collected blood samples from 48 MM patients and 48 healthy controls. Intriguingly, circBUB1B was substantially more abundant in MM patients than healthy controls (*p* < 0.001) (Fig. 4g), and the MM patients with the higher circBUB1B expression exhibited a significantly inferior EFS survival (*p* *<* 0.01) (Fig. 4h). We further performed the RNA scope assay in a tissue microarray with MM patient samples to detect the existence of circBUB1B. The results showed that the abundance of circBUB1B in relapse patient (RP) tissues was significantly higher than that in matched newly diagnosed patient (ND) and normal (NP) tissues (Fig. 4i, j), indicating that circBUB1B might be a potential biomarker for MM progression.

### CircBUB1B_544aa promotes MM cell proliferation and drug resistance

**Fig. 5:**
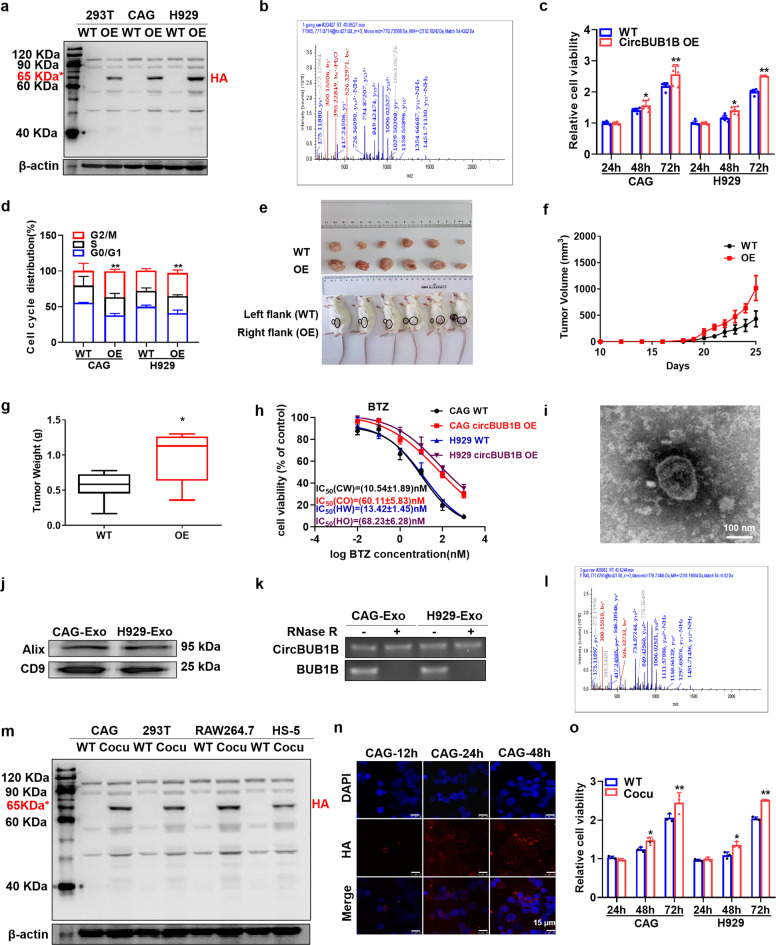
MM cells secrete circBUB1B_544aa to influence BM microenvironment and promote drug resistance. **a** WB analysis of circBUB1B_544aa OE in 293T, CAG, and H929 cells detected by HA tag antibody. **b** The specific peptides from circBUB1B_544aa were identified by mass spectrometry analysis. **c** MTT assay exhibited obviously higher cell proliferation of CAG and H929 circBUB1B-OE cells compared to WT cells. **d** Cell cycle analysis revealed that the proportion of G2/M phase significantly increased in circBUB1B-OE cells relative to WT cells. **e** Photographic images of xenograft-bearing mice were collected at day 25 and xenografts from NOD-SCID mice in the specified groups. **f** Time course of tumor growth in NOD-SCID mice. **g** Tumor weight in WT and circBUB1B-OE group at day 25 after injection of MM cells. **h** Effects of BTZ on the cell viability of CAG and H929 cells with or without circBUB1B OE. CircBUB1B-OE MM cells presented resistance to BTZ treatment. **i** Transmission electron microscopy (TEM) was used to characterize exosomes. **j** WB analysis of exosomes showed the presence of exosome marker, Alix and CD9. **k** RNA levels of circBUB1B and linear BUB1B ± RNase R were determined by RT-PCR. **l** The specific peptides from circBUB1B_544aa were identified by mass spectrometry analysis. **m** WB analysis of circBUB1B_544aa expression in CAG, 293T, RAW264.7, and HS-5 cells cocultured with CAG BUB1B-OE cells. **n** Representative confocol images for HA and DAPI showed that the CAG WT cells could dose-dependently gain circBUB1B_544aa expression and circBUB1B_544aa was mainly located in the cytoplasm. The scale bar is 15 μm. **o** MTT assay indicated obviously higher cell proliferation of co-cultured circBUB1B-OE cells compared to WT cells. The data are the mean ± SD. *p < 0.05, **p < 0.01, ***p < 0.001

To identify the function of circBUB1B_544aa, we overexpressed circBUB1B_544aa by inserting the sequence of circBUB1B_544aa into a plasmid with HA tag. Next, HA antibody and MS analysis further confirmed the specific peptide fragments from circBUB1B_544aa (Fig. 5a, b). As demonstrated in Fig. 5c, a salient increase in cell proliferation of CAG and H929 cells was provoked by elevated circBUB1B (*p* *<* 0.05). Cell cycle analysis also showed an apparent increased proportion of G2/M phase in CAG and H929 circBUB1B-OE cells (Fig. 5d). We further assessed the effect of circBUB1B on MM proliferation in vivo. CAG WT and circBUB1B-OE cells were injected subcutaneously into the right or left flanks of NOD-SCID mice, respectively. Tumors formed by circBUB1B-OE cells grew more rapidly than those formed by WT cells, with significantly increased tumor weight and volume (*p* < 0.05) (Fig. 5e–g). Similar to the exploration of BUB1B, we evaluated whether circBUB1B OE could overcome sensitivity to BTZ or ADR in MM cells. MTT assay indicated that circBUB1B-OE cells exhibited remarkably higher IC_50_ of both BTZ and ADR compared to WT cells (*p* < 0.001) (Fig. 5h, Supplementary Fig. 2a).

### MM cells secrete circBUB1B_544aa to influence the counterpart cells in BM microenvironment

It is well known that the BM microenvironment is vital to the oncogenic growth of MM cells, and many studies have been conducted to explore the impact of circular RNAs on the BM microenvironment through intercellular communication.^12^ CircRNAs are enriched and stable in exosomes that may serve as potential biomarkers for cancer detection and transfer biological activity to recipient cells.^22^ We extracted the exosomes from the culture supernatant of CAG and H929 cells, which were identified by TEM method (Fig. 5i) and WB analysis for two markers Alix and CD9 (Fig. 5j). As expected, circBUB1B was detected in the exosomes by qPCR (Fig. 5k). To examine the crucial impact of circular RNAs on the MM microenvironment through intercellular communication, we cocultured wild type of CAG, 293T, RAW, HS-5 cells with CAG circBUB1B-OE cells using transwell. We found all the cocultured cells expressing circBUB1B_544aa, as detected by HA antibody (Fig. 5m). Furthermore, MS analysis confirmed the specific peptide fragments from circBUB1B_544aa (Fig. 5l) in the cocultured cells, which indicated that the secretory circBUB1B_544aa could interfere with the adjacent or distant cells through MM microenvironment. In addition, CAG WT and circBUB1B-OE cells were cocultured for 12 h, 24 h, 48 h, and IF staining for HA and DAPI showed that the CAG cocultured cells time-dependently expressed circBUB1B_544aa (Fig. 5n). As illustrated in Fig. 5o, the proliferation rate of cocultured CAG and H929 cells was significantly increased in a time-dependent manner (*p* < 0.01) compared to the non-cocultured WT cells. MTT assay confirmed that higher IC_50_ of both BTZ and ADR were examined in cocultured CAG and H929 cells than that in WT cells (Supplementary Fig. 2b, c). In brief, we now conclude that MM cells secrete circBUB1B to influence the counterpart cells in BM microenvironment.

### CircBUB1B_544aa evokes CIN through CEP170 activation in MM cells

**Fig. 6:**
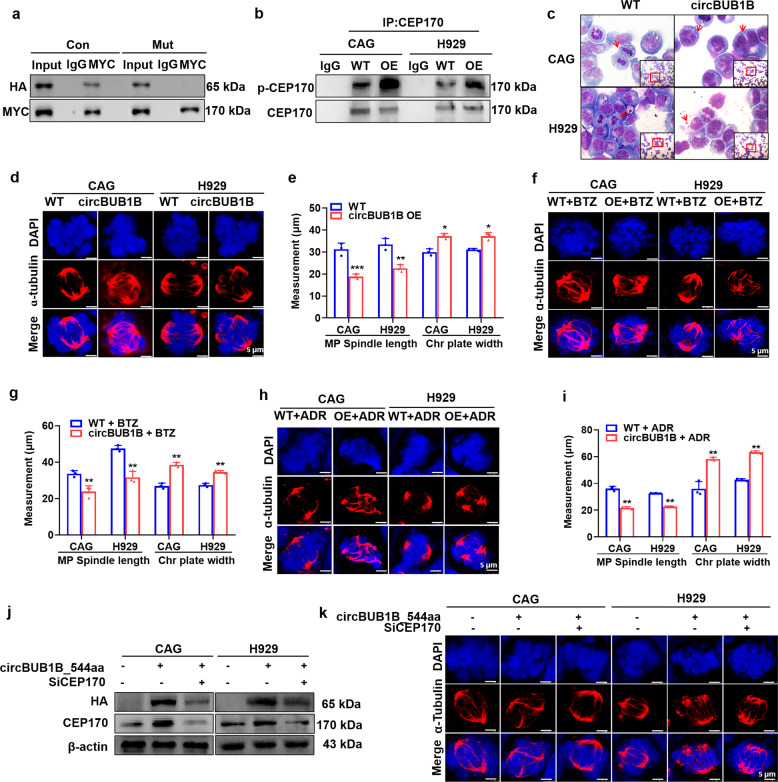
MM cells secrete circBUB1B_544aa to induce CIN. **a** Co-IP assay revealed that circBUB1B_544aa robustly interacted with native CEP170 other than mutated CEP170. **b** Co-IP assay confirmed that circBUB1B_544aa physically interacted with and phosphorylated CEP170 in circBUB1B_544aa-OE cells compared to WT cells. **c** Giemsa staining inferred that circBUB1B OE increased the separation error rate and number of multi-nuclear cells in CAG and H929 cells. **d**, **e** IF staining for α-tubulin and DAPI showed the increased chromosomal plate width and decreased mitotic bipolar spindle length in CAG and H929 circBUB1B-OE cells compared to WT cells. The scale bar is 5 μm. **f**–**i** Upon treatment with BTZ or ADR, chromosomal plate width was higher and mitotic spindle length was lower in BUB1B*-*OE cells (**f**: treating with ADR, **h**: treating with BTZ). The scale bar is 5 μm. **j** Confirmation of decreased CEP170 protein in CAG and H929 circBUB1B_544aa-OE cells upon transfection with CEP170-targeting siRNAs. **k** Targeting CEP170 by siRNA in CAG and H929 circBUB1B_544aa-OE cells significantly decreased chromosomal plate width and increased mitotic bipolar spindle length, as demonstrated by IF staining for α-tubulin and DAPI. The scale bar is 5 μm. The data are the mean ± SD. *p < 0.05, **p < 0.01, ***p < 0.001

To testify the kinase function of circBUB1B_544aa, first we confirmed that circBUB1B_544aa interacted intensely with native CEP170 using Co-IP assay, while the interaction was impaired in mutant CEP170 cells (Fig. 6a). The total phosphorylation of CEP170 was increased in circBUB1B_544aa-OE cells compared to WT cells (Fig. 6b). Giemsa staining assay demonstrated that upregulation of circBUB1B resulted in profoundly higher separation error rate and numbers of multiple nuclear cells (*p* *<* 0.01) in CAG and H929 cells (Fig. 6c, Supplementary Fig. 2d, e). Consistently, IF staining assay showed that elevated BUB1B significantly increased chromosomal plate width (*p* *<* 0.05) and decreased mitotic bipolar spindle length (*p* *<* 0.01) (Fig. 6d, e). Then we further tested whether circBUB1B_544aa OE could overcome sensitivity to BTZ or ADR in MM cells as well as BUB1B. IF staining result indicated that circBUB1B_544aa OE led to significant increments in chromosomal plate width and reductions in mitotic spindle length upon BTZ or ADR treatment in CAG and H929 cells compared to WT cells, implicating that circBUB1B_544aa was capable of inducing CIN to promote MM drug resistance as well as BUB1B (Fig. 6f–i). As to determine that CEP170 was required for the presence of CIN by interacting with circBUB1B_544aa, we knocked down CEP170 using siRNAs in circBUB1B_544aa-OE cells, as detected by WB (Fig. 6j). We found that knockdown of CEP170 in circBUB1B_544aa OE cells abrogated CIN in MM, as indicated by decreased chromosomal plate width and increased mitotic spindle length compared to non-treated circBUB1B_544aa-OE cells (Fig. 6k, Supplementary Fig. 2f). Taken together, these findings unveil that circBUB1B_544aa can also phosphorylate CEP170 to induce CIN as well as BUB1B.

### Inducible downregulation of BUB1B and circBUB1B_544aa inhibits MM cell growth in vitro and in vivo

**Fig. 7:**
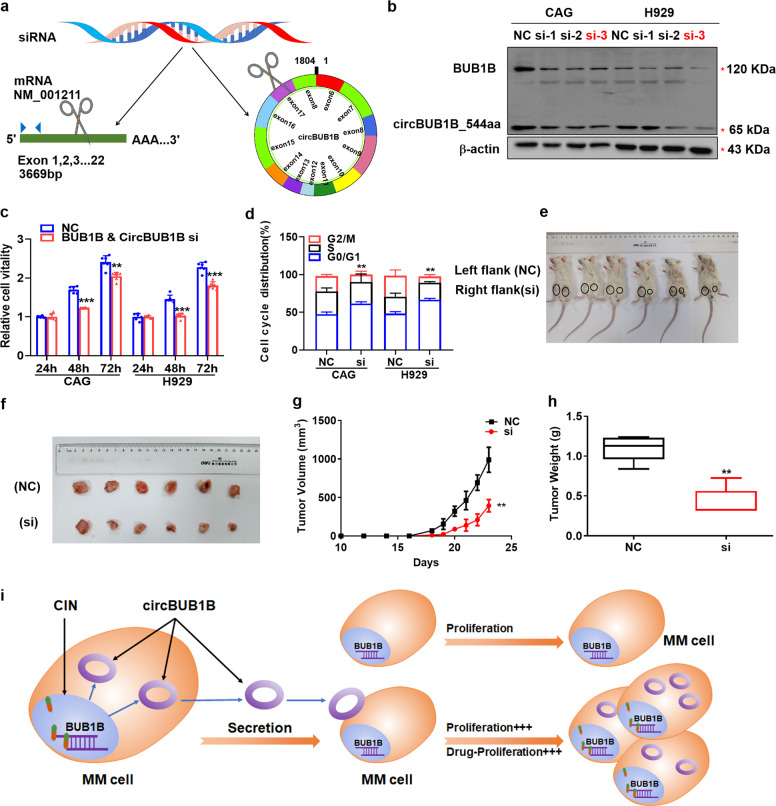
Knockdown of BUB1B and circBUB1B_544aa inhibits MM cell growth in vitro and in vivo. **a** Graphic illustration of siRNA targeting the common sequence of BUB1B and circBUB1B_544aa. **b** Confirmation of decreased BUB1B and circBUB1B_544aa in CAG and H929 cells upon transfection with siRNA-3 (si-3). **c** Reduction of BUB1B & circBUB1B_544aa prominently halted cell proliferation of CAG and H929 cells detected by MTT. **d** Cell cycle analysis demonstrated that the proportion of G2/M phase significantly decreased in BUB1B and circBUB1B cells treated with si-3 compared to NC cells. **e**, **f** Targeting BUB1B and circBUB1B_544aa by si-3 obviously inhibited tumor growth in NOD-SCID mice. **g** Time course of tumor growth in NOD-SCID mice. **h** Tumor weight in the group of silencing BUB1B and circBUB1B_544aa compared to NC group at day 23 after injection of MM cells. **i** Graphic working model illustrated that BUB1B and circBUB1B_544a*a* promoted MM malignancy by evoking CIN. The data are the mean ± SD. *p < 0.05, **p < 0.01, ***p < 0.001

To validate if BUB1B and circBUB1B_544aa were promising MM therapeutic targets, three siRNA pieces were designed to target the kinase catalytic center sequence of both the circBUB1B_544aa and its linear counterpart (Fig. 7a). WB analysis illustrated the expression of BUB1B and circBUB1B_544aa was reduced simultaneously with transfection of siRNA-3 (si-3), and the si-3 was used in the following study (Fig. 7b). As expected, MTT assay demonstrated that the proliferation of CAG and H929 cells was significantly suppressed upon blocking BUB1B and circBUB1B_544aa (*p* < 0.01) (Fig. 7c). Accordingly, BUB1B and circBUB1B_544aa si-3 cells significantly decreased in the G2/M phase relative to WT cells detected by flow cytometry (Fig. 7d). Most importantly, targeting BUB1B and circBUB1B_544aa by siRNA significantly inhibited tumor growth in NOD-SCID mice (Fig. 7e–h). Tumors formed by siBUB1B & circBUB1B_544aa cells grew more slowly than those formed by NC cells, with significantly reduced tumor weight and volume (*p* < 0.01). Summarily, these findings indicated that the newly identified circBUB1B_544aa exacerbated MM by evoking CIN and inducing drug resistance. (Fig. 7i). Targeting BUB1B and circBUB1B_544aa may afford an attractive therapeutic approach to MM.

## Discussion

MM exhibits broad gene expression alterations and cytogenetic abnormalities, which promote the progression of aggressive disease and development of drug resistance. It is well documented that CIN exhibits preeminent association with tumor malignancy and drug resistance, while in MM, our previous study has shown that CIN is strongly correlated with acquired or intrinsic drug resistance.^4,23^ Many studies have investigated the aberrant expression of BUB1B in various other cancers.^24–32^ Overexpression of BUB1B leads to aneuploidy mainly mediated by Aurora B kinase (AURKB) hyperactivation in lymphoma cells,^31^ meanwhile downregulation of BUB1B occurs in acute myeloid leukemia.^32^ Given that fine-tuning of checkpoint protein coordinative interaction is essential for reliable chromosome segregation in mitosis, both overexpression and downregulation of BUB1B can be detrimental to chromosome segregation.^33,34^ Our study demonstrated that enforcing BUB1B expression evoked CIN characterized by CGH array, shown as the significant increased multi-nuclear cells, heightened chromosomal plate width and decreased mitotic bipolar spindle length in BUB1B-OE MM cells. We identified that BUB1B directly interacted with and phosphorylated CEP170 at the Ser1260 site. CEP170 is an important centrosome amplification (CA) regulator playing a vital role in microtubule organization and microtubule stability,^35,36^ which might be a novel downstream target and signaling pathway of BUB1B. Collectively, our findings disclose that BUB1B evokes CIN so as to promote MM cell proliferation and induce drug resistance.

It has been reported that abnormal microtubule stability can trigger mitotic defects to induce CIN in cancer cells.^37^ The centrosome is the main microtubule organizing center (MTOC) and directs spindle assembly at metaphase in both meiosis and mitosis.^38^ It is consisted of a pair of centrioles and embedded pericentriolar material.^39^ Abnormal centrosome amplification (CA) leads to more than two centrosomes contributing to genomic instability in MM. CEP170, as a centrosome protein localizing in subdistal appendages of the mature centriole, was characterized by high-throughput screening of MS and MM patient cohorts in our present study. Previous studies have reported that CEP170 directly interacts with centrosome-associated kinesins KIF2A and KIF2C, and this construct acts as a key part in metaphase spindle size control.^40^ CEP170 is phosphorylated by polo-like kinase 1, and plays an important role in maintaining microtubule organization and cell morphology.^21^ Our data showed that BUB1B directly interacted with and phosphorylated CEP170 at the Ser1260 site, the mutation of which disrupted the interaction between BUB1B and CEP170, and then abolished the CIN characteristics induced by CEP170 OE. Thus, it is plausible for us to infer that BUB1B triggers CIN in MM via phosphorylating CEP170 at the Ser1260 site.

MM is highly dependent on the BM microenvironment, while circRNAs are novel RNA molecules with various biological functions and pathological significance, especially on cellular interaction in the microenvironment.^41–44^ The common function models of circRNAs are serving as miRNA sponges and interacting with associated proteins.^45^ It was recently reported that circZNF609 and circMbl were translatable.^46,47^ Unlike mRNAs, circRNAs can be translated via N6-methyladenosine modification or through internal ribosome entry site (IRES) to promote direct binding of initial factors to the circRNAs.^48–51^ Intriguingly, several circRNAs that can be translated into proteins have been confirmed to participate in tumor pathophysiology. For example, Yang et al.^45^ found the existence of FBXW7-185aa, a 21 KDa protein, which repressed glioma tumorigenesis.

In this study, we firstly identified that the circBUB1B was translatable and encoded a novel isoform, circBUB1B_544aa. Translated from the spanning junction ORF formed by the covalent connection of exon 6 and exon 8 of the BUB1B gene, circBUB1B_544aa possessed distinct amino acid sequences and contained the BUB1B kinase catalytic center compared to the linear mRNA translated proteins. Following-up exploration revealed that circBUB1B_544aa and BUB1B played a synergistic role in evoking CIN in MM through activation of CEP170, leading to MM cell proliferation and drug resistance. As reported, circRNAs might exert an important impact on the tumor microenvironment through intercellular communication, which were delivered to the microenvironment by MM cells.^52–54^ We discovered that MM cells secreted circBUB1B_544aa, which affected adjacent or distant cells in MM microenvironment. Inducible downregulation of BUB1B and circBUB1B_544aa inhibited MM cell growth in vitro and in vivo. Collectively, the BUB1B kinase catalytic center is a promising and potential therapeutic target for MM. Inhibiting this catalytic center not only inhibits MM cell proliferation and drug resistance induced by CIN, but also blocks the cellular interaction by circBUB1B in the microenvironment.

Clinical use of biomarkers displays a pivotal role at all stages of cancers, which has become one of the major approaches for cancer diagnosis and prognosis. Different from linear mRNAs, the unique covalently closed loop structures make circRNAs avoid RNaseR degradation and thus possess high stability.^55^ In addition, circRNAs are widely distributed in plasma, urine, tissue samples, cell-free saliva and other human components in a cell-specific manner.^56,57^ The expression patterns and characteristics of circRNAs, high and selective abundance, high stability, high conservation and specific expression, strongly support circRNAs serving as potential biomarkers or therapeutic targets. In present study, only 100 μL blood samples were used for examination that collected from 48 MM patients and 48 healthy controls. CircBUB1B was extremely more abundant in MM patients than healthy controls, and the MM patients with the higher circBUB1B expression exhibited a significantly inferior EFS survival. Similarly, Memczak et al. reported that circRNAs were elevated in comparable to those of linear counterparts in the blood.^58^ Meanwhile, Li et al.^59^ also found that tumor-excreted circPDE8A diffused into blood circulation by exosome transportation and plasma exosomal circPDE8A was significantly associated with tumor invasion of pancreatic ductal adenocarcinoma (PDAC) patients. Given their high tumor specificity and stability, circRNAs may become a new resource for tumor biomarkers making it an attractive target for early detection, precise treatment, and prognosis prediction.

In conclusion, our findings provide a novel and mechanistic insight of BUB1B and circBUB1B_544aa into promoting MM cell proliferation and drug resistance, which are attributed to induce CIN and activate of CEP170. BUB1B and circBUB1B_544aa are promising diagnostic markers and potential therapeutic targets in MM.

## Materials and methods

### Gene expression profiling

GEP cohorts were analyzed in the GEO database as described previously.^60,61^ The total therapy 2 (TT2 GSE2658), and the assessment of proteasome inhibition for extending remission (APEX, GSE9782) and the Dutch-Belgian Cooperative Trial Group for Hematology Oncology Group-65 (HOVON65, GSE19784) trial patient cohorts were included in analyses.

### Antibodies and reagents

Antibodies used in this study were as follows: BUB1B (4116, Cell Signaling Technology, USA), MYC (16286-1-AP, ProteinTech Group, China), CEP170 (18899-1-AP, ProteinTech Group, China), α-Tubulin (ab7291, Abcam, UK), FLAG (F-4020, Merck KGaA, Germany), β-actin (4970 S, Cell Signaling Technology), rabbit IgG (a7016) and mouse IgG (a7028, Beyotime Institute of Biotechnology, China).

Doxycycline (Dox) was purchased from the Beyotime Institute of Biotechnology (Shanghai, China). Puromycin was from Merck KGaA (Darmstadt, Germany). Bortezomib (BTZ) and Adriamycin (ADR) were purchased from Selleck Chemicals (Houston, TX). The rapid Giemsa staining kit was obtained from BBI Life Sciences (Shanghai, China).

### Cell lines and cell culture

Human MM cell lines, CAG and H929, were cultured in RPMI-1640 (Biological Industries, Israel). HEK-293 cells were cultured in DMEM (Thermo Fisher Scientific, USA). All media were supplemented with 10% fetal bovine serum (Gibco, USA), 100 U/mL penicillin, and 100 µg/mL streptomycin (HyClone, USA). All the cells were cultured at 37 ˚C in 5% CO_2_.

### RNA scope

CircBUB1B expression levels in fresh paraffin-embedded tissues were analyzed using the BaseScope^TM^ Reagent Kit v2-RED (Advanced Cell Diagnostics, Newark, CA) according to the manufacturer’s instructions. Briefly, the fresh paraffin-embedded tissues were rehydrated for 20 min, digested with protease for 40 min and incubated with the circBUB1B probes at 40 °C for 2 h. Subsequently, single amplification procedures were performed. Finally, circBUB1B expression levels were visualized with Fast Red.

### Plasmids and transfection

Plasmids containing human BUB1B cDNA and BUB1B shRNA cassettes were purchased from Generay Biotech Co., (Shanghai, China). The BUB1B coding sequence was cloned into the lentiviral vector, CD513B-1. BUB1B-targeting shRNA under the control of a DOX-inducible promoter was cloned into pTRIPZ vector. Lentiviruses were produced by co-transfection of the expression vector of interest with the packaging plasmids PLP1, PLP2, and VSVG into HEK293 cells using Lipofectamine™2000 Transfection Reagent (Invitrogen, USA).

A commercially available circular RNA expression vector PLC5-ciR (GS0104, Guangzhou Geneseed Biotech Co, China) was utilized to generate the circBUB1B-OE vector. To induce circularization, side flanking repeat sequences and SA/SD sequences were added to both sides of the 1804nt sequences (OV-circBUB1B). The front circular frame contained the endogenous flanking genomic sequences with EcoRI restriction enzyme site, and the back-circular frame contained part of the inverted upstream sequence with BamHI site. Lentiviruses were produced by co-transfection of the expression vector of interest with the packaging plasmids psPAX2 and pMD2G (Addgene) into HEK293 cells using Lipofectamine™2000 Transfection Reagent (Invitrogen, USA).

Virus supernatant was collected after 48 h. Transfected MM cells were selected by puromycin resistance. The overexpression effect was monitored by qPCR and WB.

### Cell proliferation, colony formation, and cell cycle assays

Cell proliferation rate and viability were detected by MTT assay.

For colony formation assays, clonogenic growth was determined by plating 1 × 10^4^ cells in 0.5 mL of 0.33% agar/RPMI 1640 supplemented with 10% FBS. Medium was replaced twice weekly, and cells were cultured for around 14 days. Clusters of cells were considered to be a clonogenic colony if over 40 cells were present. Colonies were imaged, and colony numbers were counted using ImageJ.

For cell cycle assays, MM cells were fixed using 70% ethanol and washed with PBS and treated with propidium iodide (PI) solution (Yeasen, China) for 30 min. Samples were analyzed using flow cytometry (Merck Millipore, Germany).

### WB and co-immunoprecipitation (Co-IP)

WB was performed as previously described.^62^ Co-IP was conducted using a Pierce Direct Magnetic IP/Co-IP kit (Thermo Scientific) following the manufacturer’s instructions.

### Immunofluorescent staining and confocal microscopy

Cells were fixed with 4% paraformaldehyde, permeabilized with PBS containing 0.1% Triton X-100, quenched with 50 mM NH_4_Cl (5 min), and blocked with 1% BSA. After overnight incubation with primary antibodies at 4 °C, slides were incubated with corresponding secondary antibodies. Images were captured using a confocal microscope (TCS SP8, Leica, Germany).

### Giemsa staining

Giemsa staining was conducted using the rapid Giemsa staining kit (BBI Life Sciences, Shanghai, China) according to the manufacturer’s instructions.

### Mass spectrometry analysis

SDS-PAGE was used to separate proteins, and gel bands at the expected size were excised and digested with sequencing-grade trypsin (Promega, USA). The resulting peptides were analyzed using a QExactive mass spectrometer (Thermo Fisher Scientific). Fragment spectra were analyzed according to the National Center for Biotechnology Information nonredundant protein database.

### MM xenografts

Wild type (WT) and BUB1B-OE cells (1 × 10^6^) were injected subcutaneously into the left and right abdominal flanks of 6~8-week old SCID/NOD mice, respectively. Then, the mice were treated with intraperitoneal (IP) administration of BTZ (1 mg/kg) or ADR (1 mg/kg) twice weekly.

Tumor diameter was measured daily using calipers. Once the tumor diameter reached 20 mm, mice were sacrificed, and the tumor tissues were collected, weighed, and photographed. All animal studies were conducted in accordance with the government-published recommendations for the Care and Use of Laboratory Animals, and were approved by the Institutional Ethics Review Boards of Nanjing University of Chinese Medicine (Ethics Registration no. 201905A003).

### Statistical analyses

Statistical analyses were performed using SPSS version 22.0 or GraphPad Prism 6.01 software, and all values were expressed as mean ± SD unless otherwise specified. A two-tailed Student’s *t*-test (2 groups) or one-way analysis of variance (ANOVA) (≥3 groups) was utilized to evaluate statistical significance. Kaplan–Meier curve and Log-rank test were employed to determine MM patient survival. *p* < 0.05 was considered statistically significant.

## Supplementary information

Supplementary_Materials

## Data Availability

The datasets used for the current study are available from the corresponding author upon reasonable request.
