# Supplementary material for: BUB1B and circBUB1B_544aa aggravate multiple myeloma malignancy through evoking chromosomal instability
Source: Signal Transduct Target Ther. 2021 Oct 7;6:361. doi: 10.1038/s41392-021-00746-6 (PMC8497505; doi:10.1038/s41392-021-00746-6)
Supplement: Supplementary file 1 — Supplementary_Materials [file 41392_2021_746_MOESM1_ESM.docx]

Supplementary Materials for

BUB1B and circBUB1B_544aa aggravate multiple myeloma malignancy through evoking chromosomal instability

Xiaozhu Tang^1,2#^, Mengjie guo^2#^, Pinggang Ding^2#^, Zhendong Deng^2^, Mengying Ke^2^, Yuxia Yuan^2^, Yanyan Zhou^2^, Zigen Lin^2^, Muxi Li^3^, Chunyan Gu^1,2*^, Xiaosong Gu^1,2,4*^, Ye Yang^2*^

Correspondence to: yangye876@sina.com; guchunyan@njucm.edu.cn; nervegu@ntu.edu.cn.

**This PDF file includes:**

Figures S1 to S2

Figure S1.


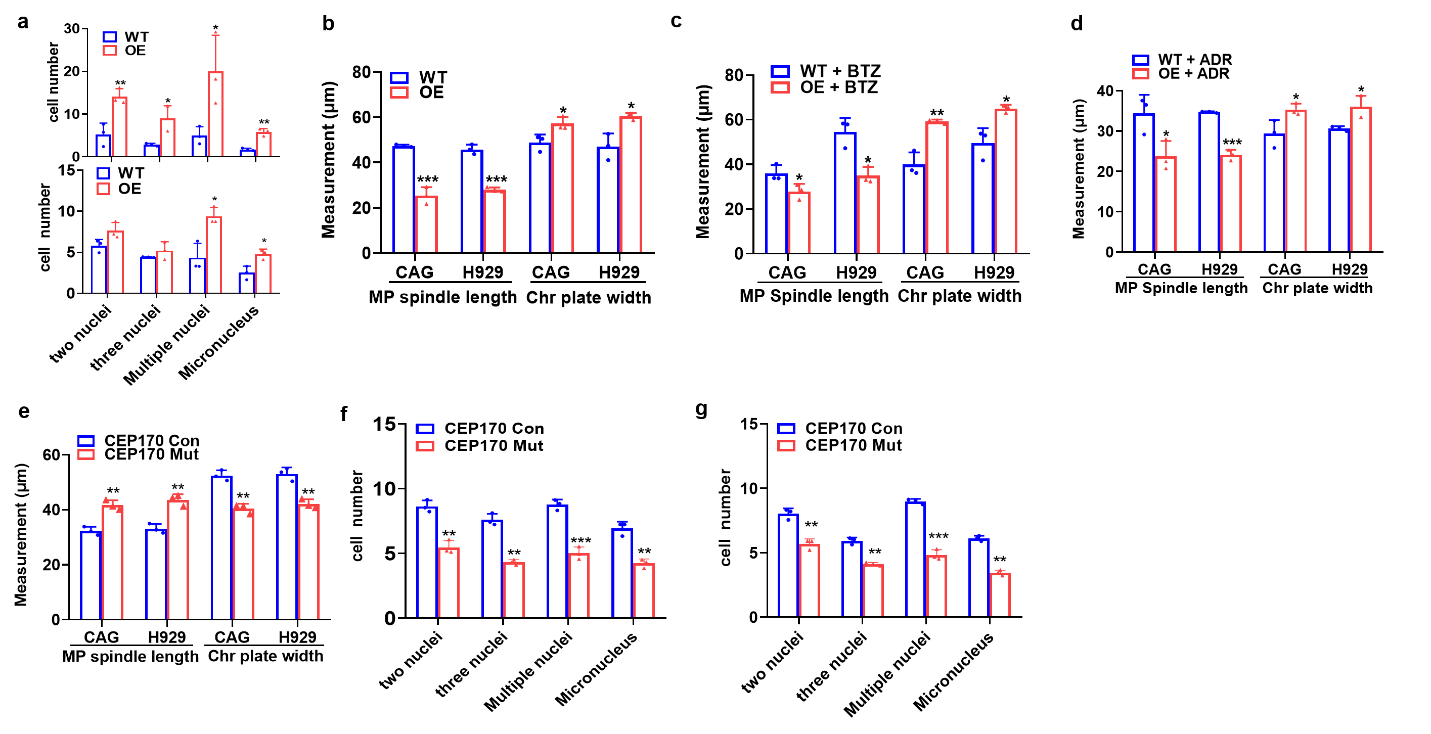


**Figure S1. BUB1B evokes chromosomal instability (CIN) in MM cells.** **a** Quantitative analysis results showed significant increased numbers of multiple nuclear cells and separation error rate in CAG and H929 BUB1B-OE cells compared to WT cells. **b** Quantitative analysis results showed significant increased chromosomal plate width and decreased mitotic bipolar spindle length in CAG and H929 BUB1B-OE cells compared to WT cells, as demonstrated by IF staining for α-tubulin and DAPI. **c,d** Upon WT and BUB1B-OE cells treated with BTZ or ADR, chromosomal plate width was significantly higher and mitotic spindle length was remarkably lower in BUB1B-OE cells with BTZ or ADR treatment than WT cells. **e** Decreased chromosomal plate width and increased mitotic spindle length were presented in mutated Ser1260Ala CEP170 cells compared to WT cells. **f,g** Overexpression of Ser1260Ala mutant CEP170 decreased the numbers of multiple nuclear cells detected by Giemsa staining compared to WT cells.

Figure S2.


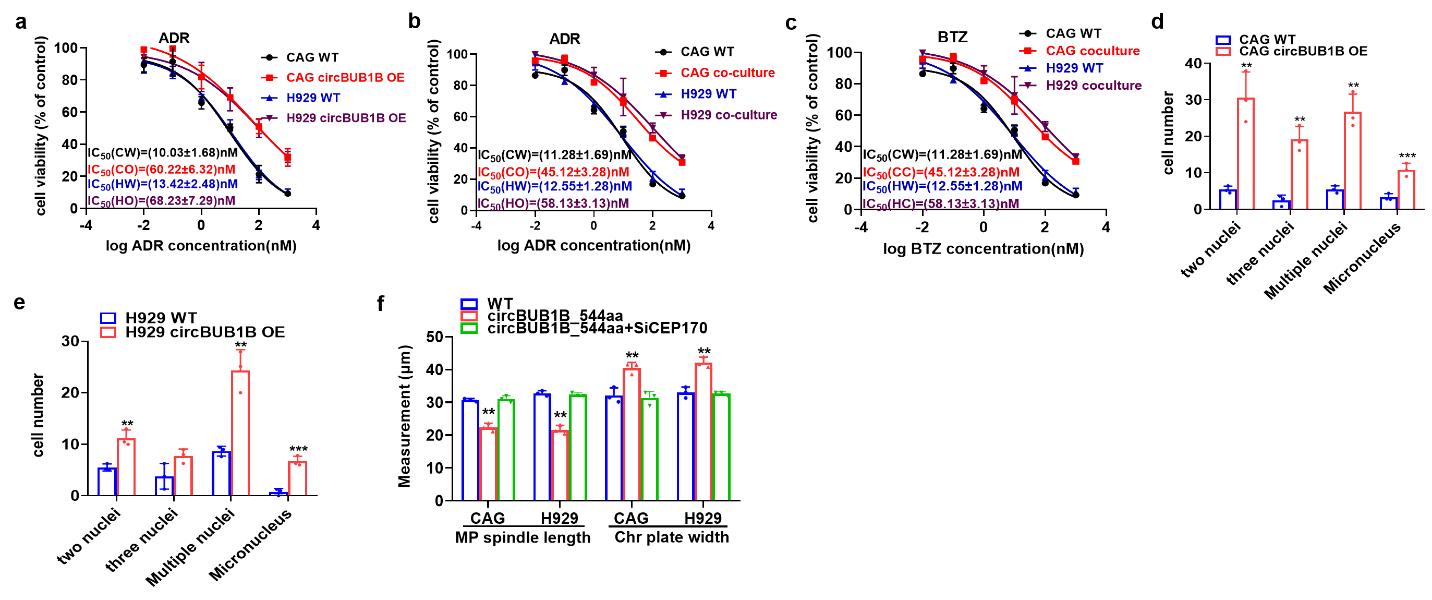


**Figure S2. MM cells secrete circBUB1B_544aa to evoke CIN in MM cells. a** MTT assay indicated that remarkably higher IC50 of both BTZ and ADR were detected in circBUB1B-OE cells than that in WT cells. **b,c** MTT assay confirmed that higher IC50 of both BTZ and ADR were examined in cocultured CAG and H929 cells than that in WT cells. **d,e** Giemsa staining demonstrated that upregulation of circBUB1B resulted in profoundly high separation error rate and numbers of multiple nuclear cells (p<0.01) in CAG and H929 cells. **f** Knockdown of CEP170 in circBUB1B_544aa-OE cells abrogated CIN characteristics indicated as decreased chromosomal plate width and increased mitotic spindle length.
